# Differential effects on human cytochromes P450 by CRISPR/Cas9-induced genetic knockout of cytochrome P450 reductase and cytochrome b5 in HepaRG cells

**DOI:** 10.1038/s41598-020-79952-1

**Published:** 2021-01-13

**Authors:** Tamara Heintze, Kathrin Klein, Ute Hofmann, Ulrich M. Zanger

**Affiliations:** 1 Dr. Margarete Fischer-Bosch Institute of Clinical Pharmacology, Stuttgart, Germany; 2 Eberhard Karls University Tuebingen, Tuebingen, Germany

**Keywords:** Biochemistry, Drug discovery, Molecular biology, Molecular medicine

## Abstract

HepaRG cells are increasingly accepted as model for human drug metabolism and other hepatic functions. We used lentiviral transduction of undifferentiated HepaRG cells to deliver Cas9 and two alternative sgRNAs targeted at NADPH:cytochrome P450 oxidoreductase (POR), the obligate electron donor for microsomal cytochromes P450 (CYP). Cas9-expressing HepaRG^VC^ (vector control) cells were phenotypically similar to wild type HepaRG cells and could be differentiated into hepatocyte-like cells by DMSO. Genetic *POR*-knockout resulted in phenotypic POR knockdown of up to 90% at mRNA, protein, and activity levels. LC–MS/MS measurement of seven CYP-activities showed differential effects of POR-knockdown with CYP2C8 being least and CYP2C9 being most affected. Further studies on cytochrome b5 (CYB5), an alternative NADH-dependent electron donor indicated particularly strong support of CYP2C8-dependent amodiaquine N-deethylation by CYB5 and this was confirmed by genetic CYB5 single- and POR/CYB5 double-knockout. POR-knockdown also affected CYP expression on mRNA and protein level, with CYP1A2 being induced severalfold, while CYP2C9 was strongly downregulated. In summary our results show that POR/NADPH- and CYB5/NADH-electron transport systems influence human drug metabolizing CYPs differentially and differently than mouse Cyps. Our Cas9-expressing HepaRG^VC^ cells should be suitable to study the influence of diverse genes on drug metabolism and other hepatic functions.

## Introduction

Application of genome editing technologies, in particular CRISPR/Cas9 to study human hepatic cytochrome P450 (CYP)-dependent drug metabolism and drug transport functions has been hampered by the limitations of the few cell models that reliably reflect relevant liver functions^1, 2^. Thus, human primary hepatocytes, often regarded as “gold standard” have a very limited life span and rapidly lose their drug metabolism and transport activities, while practically all available human hepatoma cell lines are characterized by poor liver-specific phenotype^3^. An exception are HepaRG cells, a bi-potent progenitor cell line developed from a hepatocellular carcinoma that can differentiate into either biliary or hepatocyte lineages^4^. As shown by genome-wide gene expression profiling studies, HepaRG cells are more similar to primary hepatocytes and human liver tissue than any other human liver cell line^5^. HepaRG cells demonstrate stable and functional expression of several CYP enzymes as well as phase 2 enzymes, drug transporters, and liver-specific transcription factors including dedicated ligand-activated nuclear receptors and are widely accepted as a highly useful model to study various aspects of drug metabolism, transport and its regulation^6–12^. HepaRG cells are therefore likely the best currently available human hepatic cell model to apply CRISPR/Cas9-mediated genome editing. However, application of CRISPR/Cas9 genome editing to HepaRG cells could be challenging because of their non-clonal origin and required differentiation process^4^ and to our knowledge only few studies have been reported, highlighting the difficulties with application of this method^13–16^.

Here we selected NADPH:cytochrome P450 oxidoreductase (POR) as target for our gene knockout studies in HepaRG cells. POR is a ubiquitous microsomal flavoprotein that accepts a pair of electrons from NADPH and transfers them to microsomal CYP enzymes as well as to several non-P450 enzymes, such as heme oxygenase, squalene monooxygenase or cytochrome b5 (CYB5)^17, 18^. POR thus plays a pivotal role for all microsomal CYP-catalyzed oxidative metabolic conversions of numerous endogenous and exogenous substrates including most drugs, as well as cholesterol and lipid homeostasis and other physiological processes. The various CYP isoenzymes and their electron donors POR and CYB5 are believed to dynamically associate to form functional complexes involving protein–protein and protein–lipid interactions that hereby influence P450 catalytic function and efficiency^19–21^. In contrast to the multigenic mammalian *CYP* superfamily, *POR* is encoded by a single gene, which was shown to be essential for early stage development as germline deletion of *Por* in mice leads to embryonal death around day 13 due to severe disturbances in retinoid homeostasis^22, 23^.

Conditional *Por* deletion in mouse liver results in phenotypically normal and fertile mice with profoundly decreased hepatic microsomal Cyp-function, reduced circulating cholesterol and triglyceride levels, as well as hepatic lipidosis^24–26^. Residual Cyp activities in the absence of Por indicated that other electron donating systems, especially the cytochrome b5 (Cyb5)/Cyb5 reductase system contribute and may even suffice in some cases^27^. On expression level, some hepatic CYP proteins were found to be increased, a compensatory response ascribed to accumulated eicosanoids, retinoids, fatty acids or steroid hormones that serve as ligands to nuclear receptors and other transcriptional regulators. In humans, POR is expressed at five to tenfold lower stoichiometric level in liver compared to total CYP content and the possibility that it may be a limiting factor for CYP activity has been discussed^28–32^. Early studies used siRNA to knockdown POR in human and rat hepatocytes^33, 34^. In more recent studies CRISPR/Cas9-induced genome editing was used in Hepa1c1c7 cells for *POR*-knockout to investigate mechanisms of benzo[a]pyrene resistance^35, 36^. There are several other studies that investigated diminished POR in cellular models. CRISPR/Cas9 or zinc-finger induced knockout or shRNA knockdown of POR was used for the investigation of the role of POR as a predictive biomarker for hypoxia activated prodrug sensitivity^37–40^. However, to our knowledge none of the studies on *POR*-knockout in cell models studied its effects on human drug metabolizing CYPs. Similarly, the importance of the CYB5/CYB5 reductase system as alternative electron donor for human drug metabolism has been studied over the years in many systems, including human liver microsomes^41^, reconstitution of recombinant enzymes^42^, structural interaction models^43, 44^, and humanized mouse models^45, 46^, yet its presence and role in HepaRG cells has not been studied to our knowledge so far.

Here we used lentiviral transduction to establish Cas9-expressing HepaRG cells and found that they retain common characteristics and differentiation potential of wild type HepaRG. Using lentiviral transduction we show highly efficient and permanent reduction of POR expression, enabling us to study its effects on different microsomal CYP activities as well as their expression. Since the effects were surprisingly different between CYPs, we also studied the role of CYB5 by transient knockdown, which revealed also differential and partially compensatory influences on different CYP activities. Our data confirm and extend our understanding of the complex CYP/POR/CYB5 drug metabolism system and they show that HepaRG can be a versatile tool to study the role of diverse genes on hepatic functions in a metabolically competent human hepatic cell line.

## Results

### Genotyping of HepaRG cells

According to Gripon et al.^4^, HepaRG cells carry an additional chromosome 7, which however harbours a deletion that includes the location of the *POR* gene. Thus, HepaRG cells are diploid for *POR*, but to our knowledge, no information on *POR* genotype in HepaRG cells has been published. We therefore sequenced the entire *POR* gene including all exons and adjacent intron regions, resulting in genotype POR*1/*37. In contrast to the common allele *28 (A503V), which can influence CYP activities in diverse ways, *37 (A503V + V631I) is a rare allele (< 1%) that has not been functionally characterized (www.PharmVar.org)^31, 47, 48^. As *POR*-knockout effects on CYP-activities may also depend on *CYP* genotype, we determined major alleles for the *CYP*s included in our study (Table 1). While previously described genotypes for CYPs *2C9* (*2/*2), *2C19* (*1/*1), *2D6* (*2/*9) and *3A5* (*3/*3) were verified^49^, we found that HepaRG cells are homozygous for CYP2C8*3 and heterozygous for CYP2B6*6 (Table 1).

**Table 1:** Genotypes of *POR, CYP1A2, CYP2B6, CYP2C8, CYP2C9, CYP2C19, CYP2D6* and *CYP3A4* in HepaRG cells.

Gene	Genoype	Phenotype of variant alleles	Method
*POR*	*1/*37	Not known^31^	Sequencing
*CYP1A2*	*1/*1F	Higher inducibility	Sequencing
*CYP2B6*	*1/*6	Decreased function	OpenArray^a^
*CYP2C8*	*3/*3	Increased in vitro function^59^	Sequencing
*CYP2C9*	*2/*2	Decreased function	OpenArray^b^
*CYP2C19*	*1/*1	No variant allele detected	OpenArray^c^
*CYP2D6*	*2/*9	Decreased function (*9)	Sequencing
*CYP3A4*	Not *1B, not *22	No variant allele detected	OpenArray^d^
*CYP3A5*	*3/*3	Splicing defect, severely decreased expression	OpenArray^e^

Predesigned Taqman assays:

^a^AHFBATH, C__60732328_20, AHABIR9.

^b^C__27104892_10, C__25625805_10, C__30634132_70, C__27859817_40.

^c^C____469857_10, C__25745302_30, C__30634136_10, C__30634130_30, C__25986767_70 C__27861809_10, C__27861810_10, C__30634128_10, C__27531918_10.

^d^C___1837671_50, C__59013445_10.

^e^C__30203950_10, C__32287188_10, C__26201809_30.

### Lentiviral transduction of HepaRG cells

**Figure 1:**
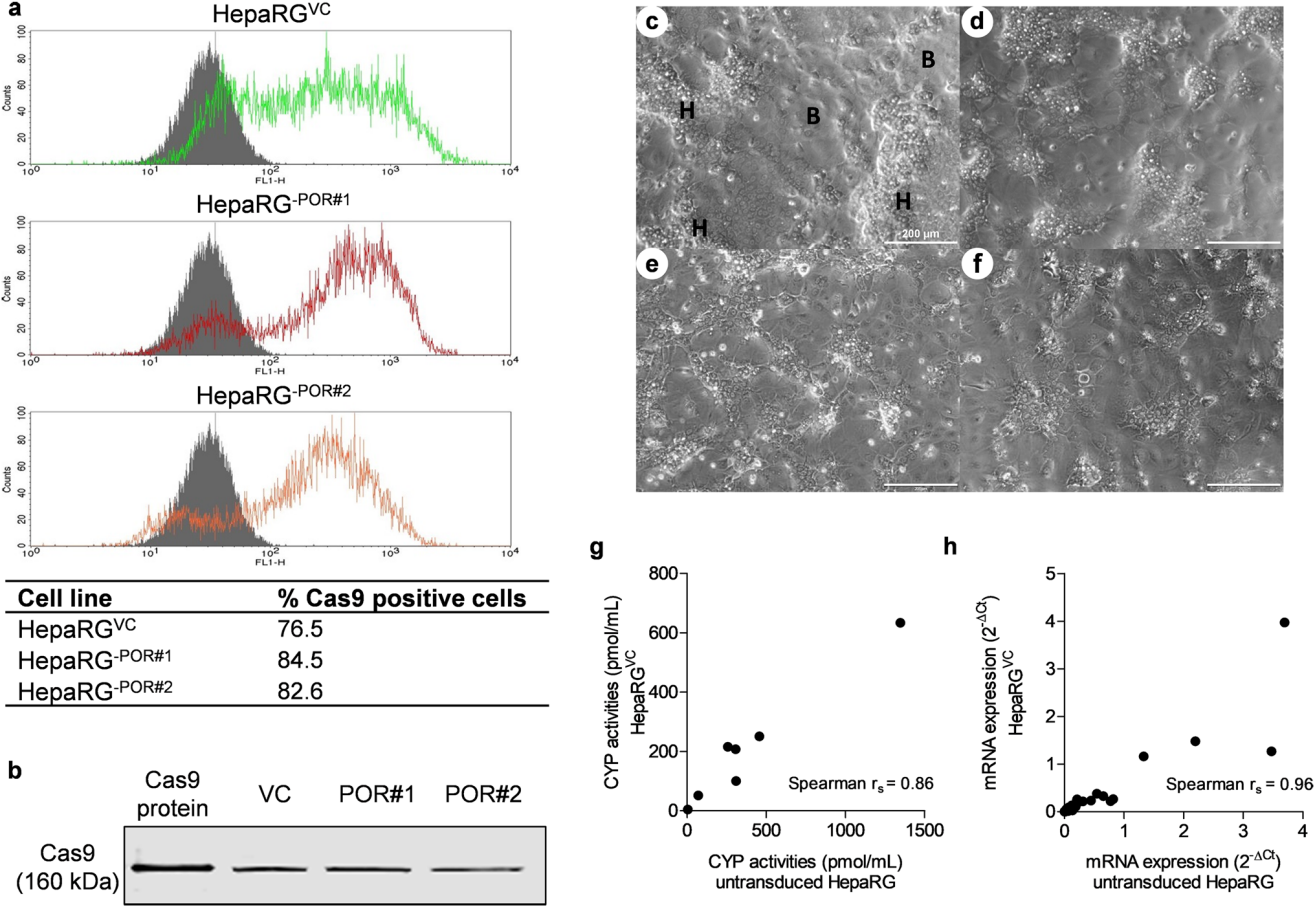
Cas9 Expression in transduced HepaRG cells. (**a**) Flow cytometry analysis of undifferentiated HepaRG^VC^ (green), HepaRG ^–POR#1^ (red) and HepaRG^-POR#2^ (orange) in comparison to untransduced HepaRG cells (grey) (**b**) Western blot analysis of Cas9 expression in lysates of undifferentiated HepaRG^VC^, HepaRG ^–POR#1^ and HepaRG^-POR#2^, Cas9 protein as positive control (see Supplementary Fig. S1 online for full blot). (**c-f**) Morphology of untransduced, differentiated HepaRG cells. H: hepatocyte-like cells; B: biliary-like cells (**c**) compared to differentiated HepaRG^VC^ (**d**), HepaRG ^–POR#1^ (**e**) and HepaRG^-POR#2^ (**f**). (**g**) Correlation of 7 CYP activities in transduced versus untransduced differentiated HepaRG cells. (**h**) Correlation of mRNA expression levels of 72 genes in transduced versus untransduced differentiated HepaRG cells.

As HepaRG cells are difficult to transfect with lipofection or nucleofection methods^50^, we used lentiviral transduction of undifferentiated cells for delivery of Cas9 and sgRNAs. The high transduction efficiency coupled with antibiotic selection lead to a high proportion of cells (> 75%) expressing Cas9 (Fig. 1a,b). We next examined whether transduced HepaRG^VC^ cells are still able to differentiate with DMSO into hepatocyte-like cells. Indeed, using standard differentiation conditions, Cas9-expressing HepaRG^VC^ cells were morphologically comparable to wild type HepaRG cells with respect to their ability to differentiate into hepatocyte-like cells and biliary cells (Fig. 1c–f). Moreover, enzyme activities simultaneously determined for seven CYPs correlated strongly (r_S_ = 0.86) with those of wild type cells, although the absolute activities tended to be somewhat lower (Fig. 1g). Analysis of a broader set of genes showed also a tendency to lower expression in HepaRG^VC^ versus HepaRG, but confirmed highly similar gene expression patterns (r_S_ = 0.94; Fig. 1h). Taken together these findings suggested that HepaRG^VC^ cells retained the most important characteristics of HepaRG and should thus be highly suitable for genome editing.

### Characterization of POR-knockout

**Figure 2:**
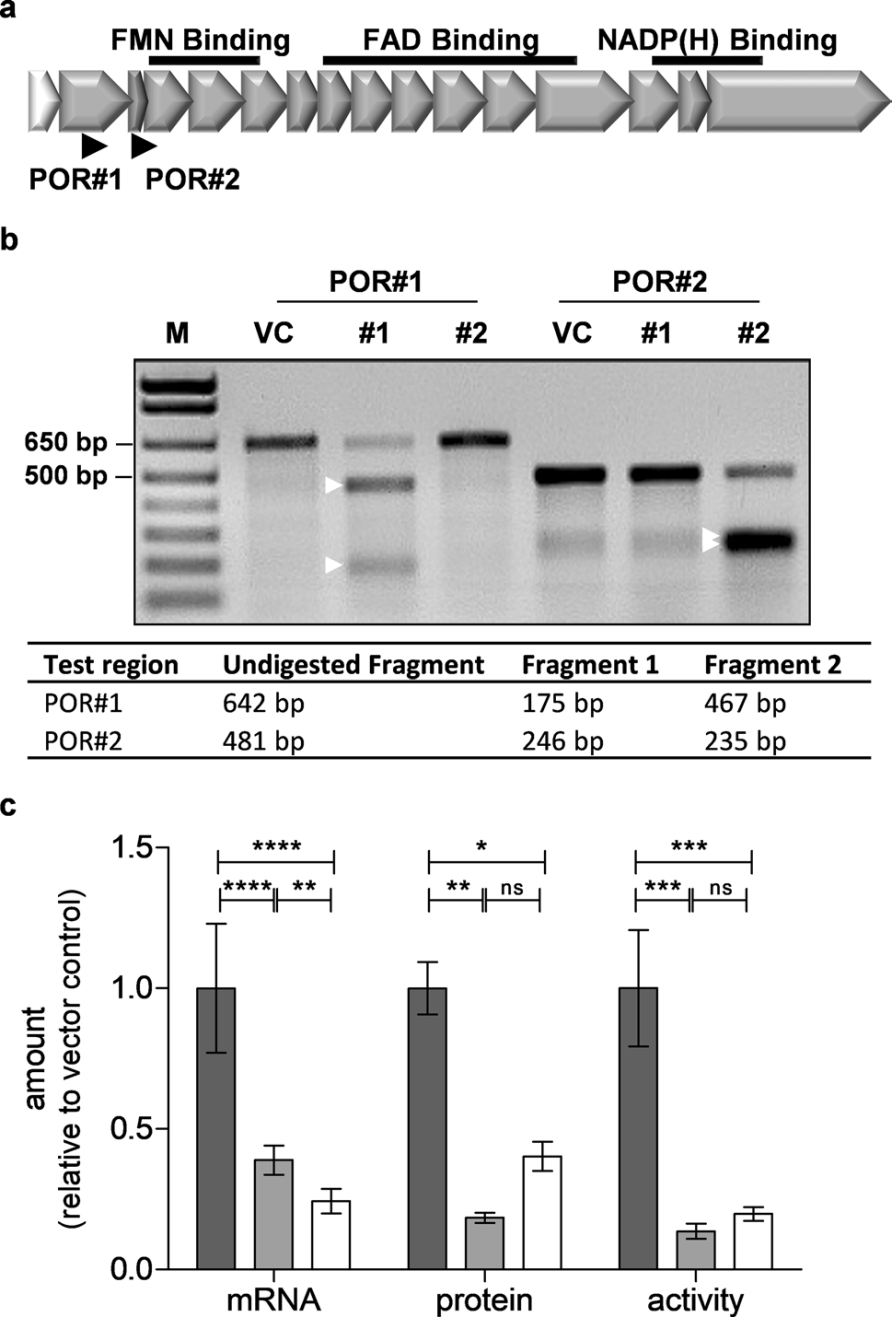
Validation of genetic *POR*-knockout in transduced HepaRG cells. (**a**) Location of *POR*-targeting gRNAs relative to exon structure (gene chr7:75,899,200–75,986,855) indicating one 5′-untranslated exon (white) and 15 translated exons as well as binding regions (black) for FAD, FMN, and NADPH. The positions of two sgRNAs targeting exon 2 (POR#1) or the FMN binding site in exon 4 (POR#2) are indicated by arrows. (**b**) T7E1 digest of DNA of transduced HepaRG cells. M: DNA marker; lanes 2–4: digest products of test region POR#1; lanes 5–7: digest products of test region POR#2; VC: vector control; #1: sgRNA POR#1; #2: sgRNA POR#2. Digested fragments are marked with white arrows. The expected fragment sizes are summarized below. (**c**) POR expression and cytochrome c reductase activity in transduced differentiated HepaRG cells. POR mRNA was quantified in cell lysates and POR protein and cytochrome c reductase activity were quantified in microsomes. Mean levels ± SD are shown relative to vector control (VC) set at 1 (dark grey: VC, light grey: POR#1, white: POR#2). Statistical significance was assessed by unpaired t-test (*p < 0.05, **p < 0.01, ***p < 0.001, ****p < 0.0001; ns, not significant).

For CRISPR/Cas9-mediated knockout of *POR* in HepaRG cells we designed one sgRNA (POR#1) near the 5′-end of exon 2 using the common CHOPCHOP tool (Fig. 2a). A previously reported sgRNA (POR#2), which binds near the 5′-FMN binding site in exon 4, was simultaneously analyzed for comparison^39^. Predicted CRISPR/Cas9 editing was validated for both sgRNAs using the T7E1 assay (Fig. 2b). Whereas both sgRNAs had comparable efficiency scores (51.6 and 52.6, respectively), sgRNA POR#2 was predicted to bind to three off-targets. However, gene editing in these regions could be excluded by T7E1 assay.

Analysis of POR in differentiated HepaRG cells revealed that both sgRNAs were similarly effective and reduced POR mRNA and protein by 60 to 80% (Fig. 2c). Although there were minor differences in reduction of mRNA and protein between sgRNA POR#1 and POR#2, they were not consistent and we thus consider them as experimental variability. Determination of POR-mediated cytochrome c reductase activity in microsomes of differentiated HepaRG cells revealed up to 90% decrease for both sgRNAs, in good agreement with the effects on mRNA and protein level. It should be noted that the residual POR expression and activity is the result of a mixture of individual cells with homozygous, heterozygous and no genetic knockout of *POR*. Therefore, the phenotype of these cells is a POR knockdown rather than a complete knockout. For comparison, the difference in POR activity between undifferentiated and differentiated HepaRG microsomes was 0.006 ± 0.0009 U/mg versus 0.029 ± 0.006 U/mg, respectively, and a similar value was obtained in a pool of human liver microsomal fractions (0.034 ± 0.004 U/mg).

### Impact of POR-knockdown on CYP enzyme activities

**Figure 3:**
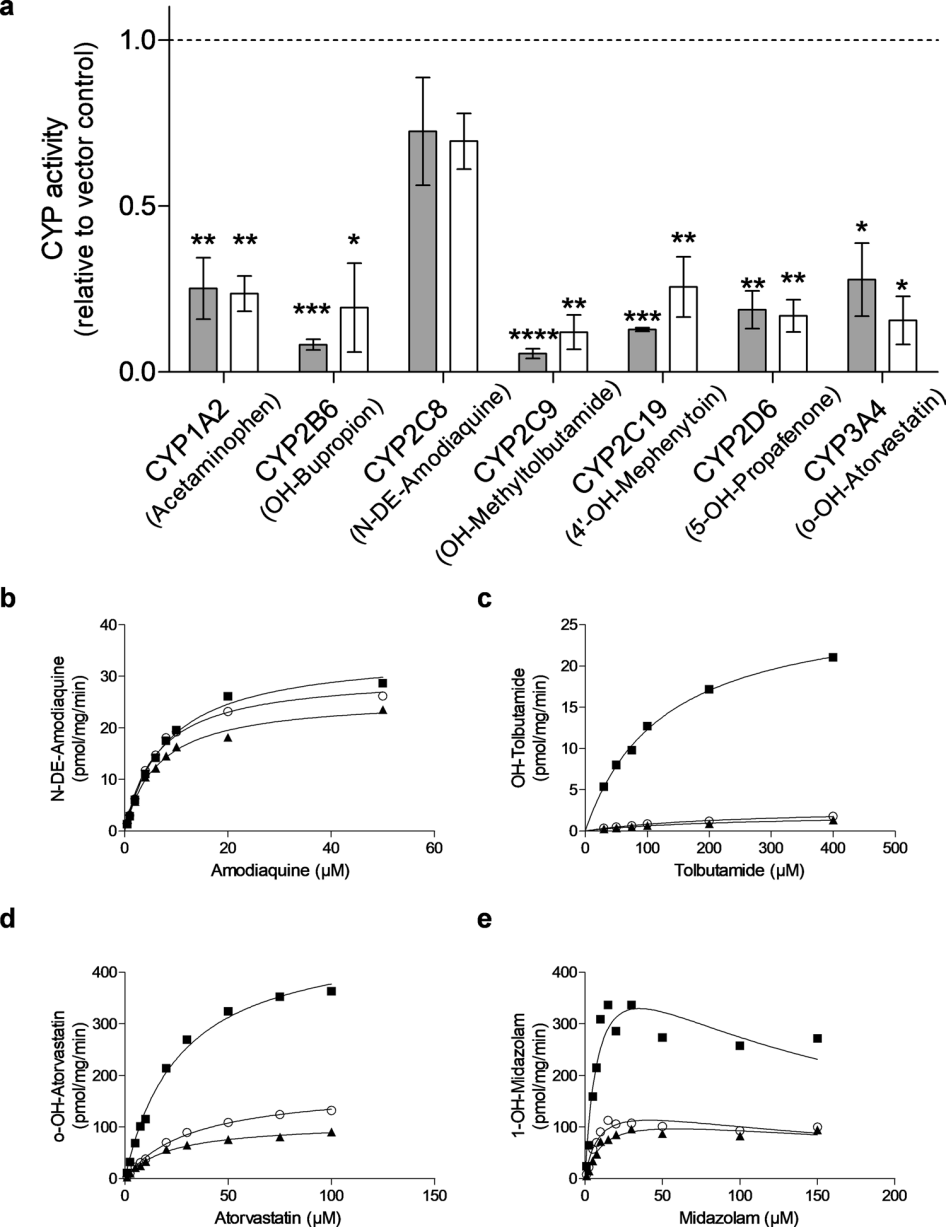
CYP activities in microsomal fractions of HepaRG^-POR^ cells. (**a**) HepaRG cells transduced with sgRNA POR#1 (grey) or POR#2 (white) were differentiated for 3 weeks and harvested for microsome preparation. Enzyme activities of seven CYP enzymes were determined simultaneously by cocktail LC–MS/MS assay and given relative to HepaRG^VC^. Results are shown as means ± SD of four independent experiments. Statistical significance unpaired t-test (*p < 0.05, **p < 0.01, ***p < 0.001, ****p < 0.0001). (**b-e**) Kinetic analysis of selected substrate conversions in HepaRG microsomes. HepaRG cells transduced with vector control (VC, filled square), sgRNAs POR#1 (filled triangle) and POR#2 (open circle) were differentiated for 3 weeks and harvested for microsome preparation; (**b**) amodiaquine (CYP2C8); (**c**) tolbutamide (CYP2C9); (**d**) atorvastatin (CYP3A4); (**e**) midazolam (CYP3A4). Data were analyzed by Michaelis–Menten model (**b**–**d**) or by substrate inhibition model (**e**).

We used our previously established CYP cocktail LC–MS/MS assay to measure enzyme activities of seven CYPs simultaneously in microsomes isolated from both types of HepaRG^-POR^ and HepaRG^VC^ cells. As shown in Fig. 3a, all CYP activities except for amodiaquine N-deethylation were negatively affected by both sgRNAs. While the effects on individual CYP isoforms differed substantially, the pattern was similar for both sgRNAs. Across all CYPs measured, we observed 1.4- to 20-fold reductions in enzyme activity. The strongest effect of *POR*-knockdown was observed for CYP2C9 (85–95% reduction of activity), while no significant effect was seen for CYP2C8. Compared to other CYP activities, CYP2C8-catalyzed amodiaquine N-deethylation thus appeared to be rather insensitive against variable POR levels.

In order to characterize these changes in more detail we performed kinetic experiments exemplarily for CYP2C8, CYP2C9 and CYP3A4 using the substrates amodiaquine, tolbutamide, and atorvastatin, respectively, and midazolam as a second CYP3A4 substrate (Fig. 3b–e, Table 2). The effects of *POR*-knockdown on kinetic parameters of amodiaquine N-deethylation were again surprisingly small, reducing V_max_ by only 26% (POR#1) and 13% (POR#2), while substantial reductions in V_max_ were found for the other substrates (Table 2). Only for tolbutamide an increase in K_M_ (~ twofold) was observed, effectively reducing intrinsic clearance for CYP2C9 to about 5% in HepaRG^-POR^ compared to HepaRG^VC^ cells. The kinetic measurements of the conversion of atorvastatin and midazolam by CYP3A4 showed both an approximately fourfold decrease of V_max_ while K_M_ was not consistently affected.

**Table 2:** Calculated kinetic parameters of selected substrate conversions.

	V_max_ [95% CI] (pmol/mg/min)	K_M_ [95% CI] (µM)	K_i_ [95% CI] (µM)	Cl_int_ [95% CI]
**Amodiaquine**				
VC	34.7 [31.5 to 37.9]	8.3 [6.3 to 10.2]		4.2 [3.7 to 5.0]
POR#1	25.8 [23.7 to 27.9]	6.5 [5.0 to 7.9]		4.0 [3.5 to 4.7]
POR#2	30.3 [27.8 to 32.9]	6.4 [4.9 to 7.8]		4.7 [4.2 to 5.7]
CYP2C8 R	26.1 [23.4 to 28.8]	4.2 [2.7 to 5.8]		6.2 [5.0 to 8.7]
CYP2C8 LR	2.0 [1.8 to 2.1]	3.5 [2.6 to 4.5]		0.57 [2.5 to 0.69]
**Tolbutamide**				
VC	27.7 [25.6 to 29.8]	125 [104 to 148]		0.22 [0.20 to 0.25]
POR#1	2.1 [1.5 to 2.7]	235 [106 to 364]		0.009 [0.007 to 0.014]
POR#2	2.8 [2.2 to 3.3]	240 [147 to 332]		0.01 [0.001 to 0.015]
CYP2C9 R	65.5 [39.4 to 91.7]	817 [381 to 1253]		0.08 [ 0.07 to 0.10]
CYP2C9 LR	3.0 [2.6 to 3.3]	214 [170 to 258]		0.01 [0.013 to 0.015]
**Atorvastatin**				
VC	476 [433 to 520]	26.3 [20.1 to 32.5]		18.1 [16.0 to 21.5]
POR#1	108 [99.4 to 116]	21.4 [16.8 to 26.0]		5.0 [4.5 to 5.9]
POR#2	179 [166 to 192]	33.1 [27.5 to 38.7]		5.4 [5.0 to 6.0]
CYP3A4 R	5.7 [4.9 to 6.5]	34.4 [23.0 to 45.8]		0.17 [0.14 to 0.21]
CYP3A4 LR	2.2 [1.9 to 2.5]	27.5 [17.8 to 37.2]		0.08 [0.07 to 0.11]
**Midazolam**				
VC	499 [233 to 766]	9.0 [0.0 to 19.3]	137 [0 to 329]	55.4 [0 to 39.7]
POR#1	136 [79.0 to 193]	12.1 [1.7 to 22.4]	286 [0 to 727]	11.2 [8.6 to 46.5]
POR#2	162 [98.2 to 226]	9.0 [1.2 to 16.7]	191 [0 to 424]	18.0 [13.5 to 81.8]
CYP3A4 R	1.2 [0.93 to 1.5]	2.7 [0.81 to 4.6]	231 [0 to 531]	0.44 [0.33 to 1.2]
CYP3A4 LR	1.0 [0.68 to 1.4]	3.5 [0.43 to 6.6]	202 [0 to 511]	0.29 [0.21 to 1.4]

**Figure 4:**
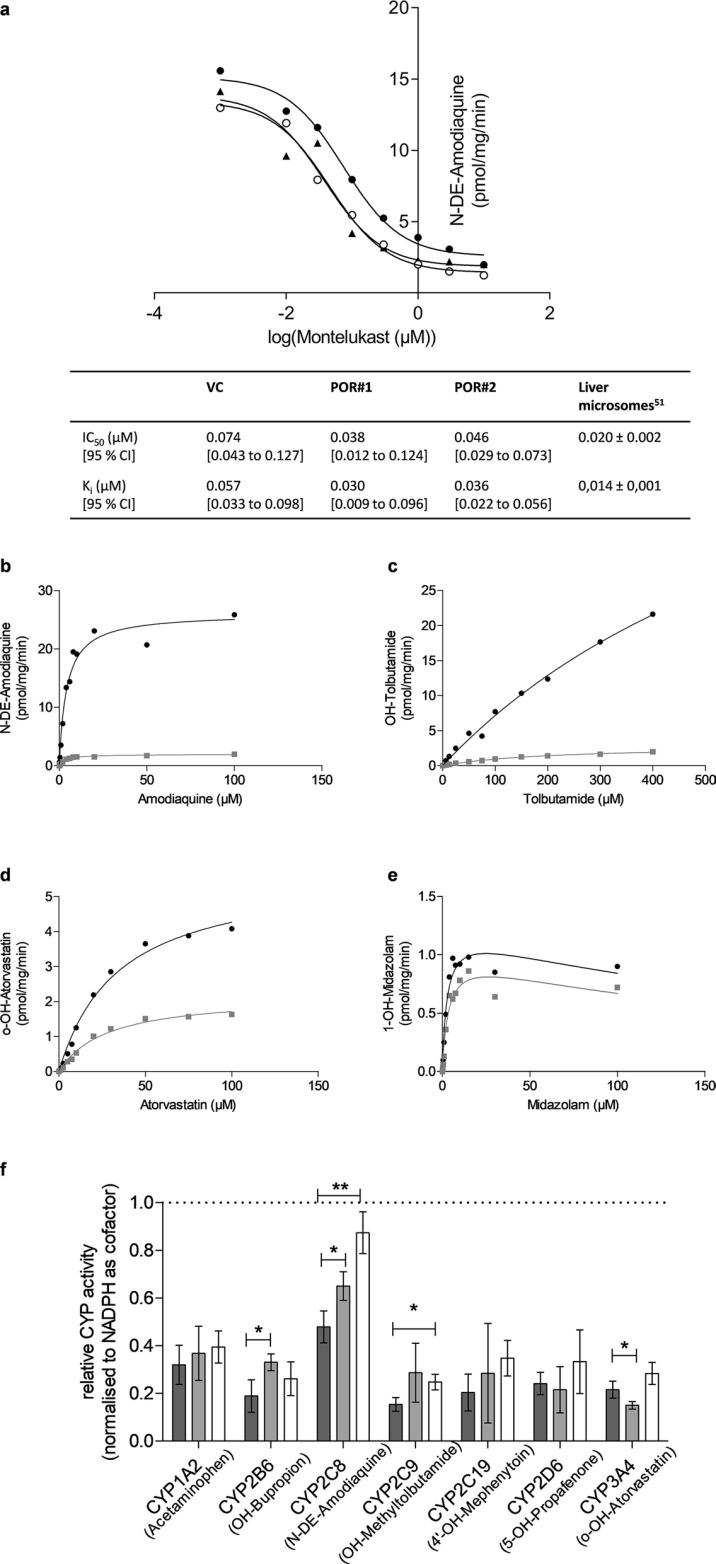
In depth analysis of CYP2C8-mediated amodiaquine N-deethylation. (**a**) Inhibition of amodiaquine N-deethylation with montelukast in microsomal protein. HepaRG cells transduced with vector control (VC, filled circle), sgRNAs POR#1 (filled triangle) and POR#2 (open circle) were differentiated for 3 weeks and harvested for microsome preparation. Inhibition parameters IC_50_ and K_i_ were determined by one site competition model. (**b**–**e**) Kinetic analysis of selected substrate conversions in bactosomes containing recombinant CYP enzymes coexpressed with high (filled circle) or low levels filled square) of POR: (**b**) amodiaquine (CYP2C8); (**c**) tolbutamide (CYP2C9); (**d**) atorvastatin (CYP3A4); (**e**) midazolam (CYP3A4). Data were analyzed by Michaelis–Menten model (**b**–**d**) or by substrate inhibition model (**e**). (**f**) Relative CYP-activities in microsomal preparations of differentiated HepaRG cells transduced with VC (dark grey), sgRNA POR#1 (light grey) and POR#2 (white) with either NADPH (set to 1.0) or NADH as cofactors. Results are means ± SD of 4 independent experiments. Statistical significance was assessed by unpaired t-test (*p < 0.05, **p < 0.01).

Substrates were incubated at different concentrations with microsomal fractions of HepaRG cells transduced with vector control (VC) or sgRNA POR#1, POR#2 or with bacterial membrane vesicles containing recombinant CYP2C8, CYP2C9 and CYP3A4 coexpressed with high (R) or low (LR) levels of POR. Following metabolite quantification by LC–MS/MS, kinetic parameters K_M_ and V_max_ were determined by Michaelis–Menten model or by substrate inhibition model, and K_i_ was determined by one site competition model. Internal clearance (Cl_int_) was calculated using this equation: ${\mathrm{Cl}}_{\mathrm{int}}=\frac{{\mathrm{V}}_{\mathrm{max}}}{{\mathrm{K}}_{\mathrm{M}}}$. Results are shown with 95% CI given in brackets. See Figs. 3 and 4 for further details.

### In depth analysis of CYP2C8-mediated amodiaquine N-deethylation

As the striking insensitivity of amodiaquine N-deethylation towards *POR*-knockdown was surprising, we made additional analyses. Using the potent and specific CYP2C8 inhibitor montelucast^51^ we confirmed similarly strong inhibition of amodiaquine N-deethylation in HepaRG^VC^ as well as in both types of HepaRG^-POR^ microsomes (Fig. 4a), suggesting catalysis by CYP2C8.

To test POR sensitivity of amodiaquine N-deethylation in a different system we next used commercially available bactosomes containing recombinant CYP2C8, CYP2C9 and CYP3A4 coexpressed with high or low levels of POR (Fig. 4b–e). Indeed, in this system amodiaquine N-deethylation was clearly POR sensitive, suggesting that CYP2C8 may be supported by an alternative electron donor in HepaRG cells.

As a primary candidate we suspected the CYB5/CYB5 reductase system, which is lacking in the bacterial membrane vesicles but should be present in HepaRG cells^52^. Taking advantage of the fact that CYB5/CYB5 reductase depends on NADH rather than on NADPH, we performed comparative activity measurements in HepaRG^VC^ and both types of HepaRG^-POR^ microsomes. All seven CYP activities could be supported by NADH alone, albeit at a lower level (~ 15–50% compared to NADPH; Fig. 4f). All differences of NADH- versus NADPH-supported activities were statistically significant (*p* < 0.05; two-way ANOVA with Bonferroni correction). Interestingly, CYP2C8 activity was least affected by the cofactor change (50%) and in HepaRG^-POR^ microsomes NADH-dependent activity was significantly increased (POR#1, 65%; POR#2, 87%). Taken together these data suggested involvement of CYB5 in the various CYP activities, which seemed to be particularly strong for CYP2C8. To prove this assumption directly we created genetic *CYB5A* single- and *POR/CYB5A* double-knockout cells.

### Effects of genetic CYB5A and combined POR/CYB5A knockout on CYP enzyme activities

**Figure 5:**
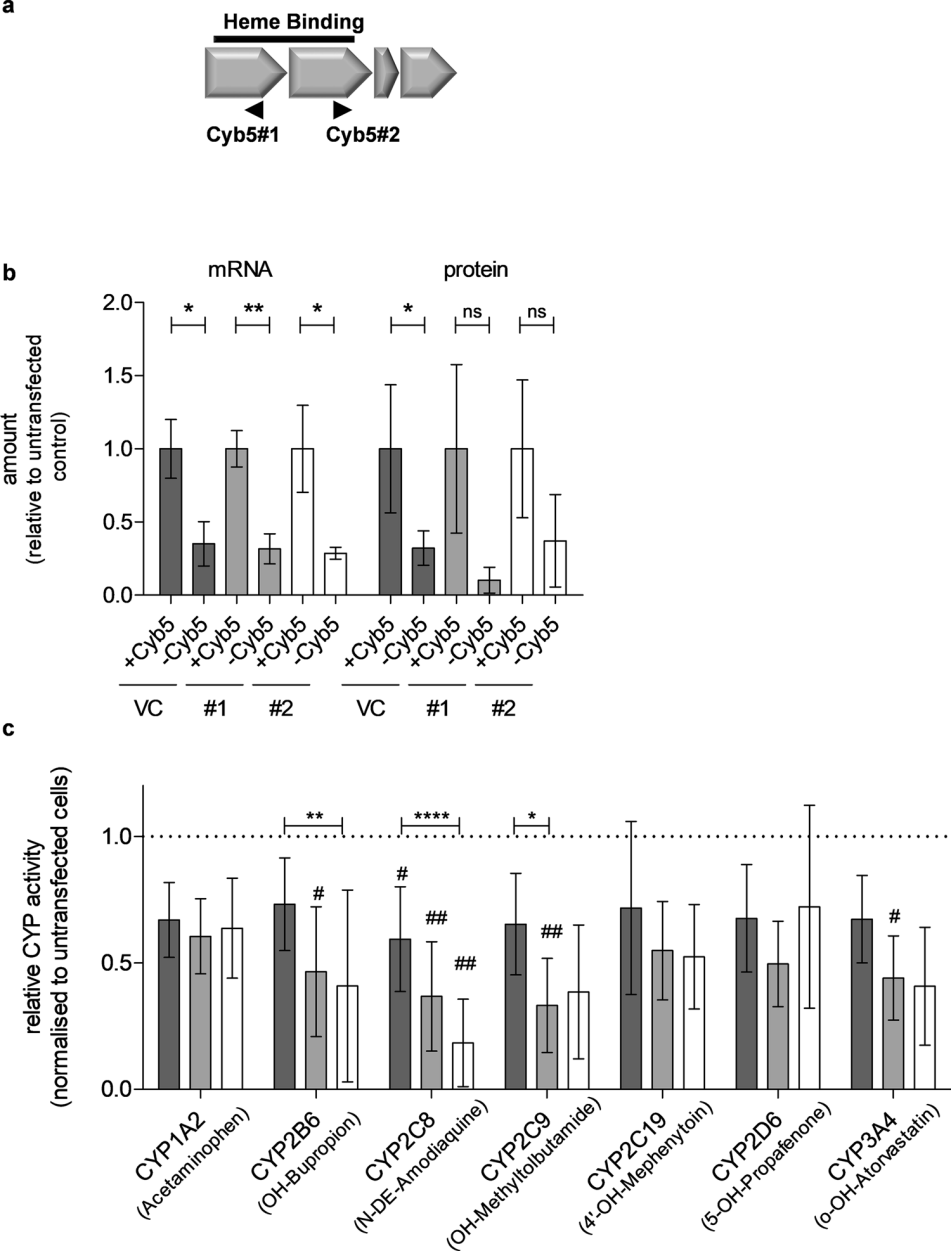
Genetic *CYB5A* and *POR/CYB5A* double-knockout in transduced HepaRG cells. (**a**) Location of *CYB5A*-targeting gRNAs relative to exon structure (gene chr18:74,250,846–74,292,016) indicating 4 translated exons as well as the binding region (black) for heme. The positions of two sgRNAs targeting exon 1 (CYB5#1) or exon 2 (CYB5#2) are indicated by arrows. (**b**) CYB5 mRNA and protein expression in transduced differentiated HepaRG cells quantified in cell lysates. Mean levels are shown relative to vector control (VC) set at 1 with SD bars (dark grey: VC, light grey: POR#1, white: POR#2). Results are means ± SD of three independent experiments. Statistical significance was assessed by unpaired t-test (**c**) Enzyme activities of seven CYP enzymes were determined simultaneously by cocktail LC–MS/MS assay in VC (dark grey), sgRNA POR#1 (light grey) and POR#2 (white) cells. Results are means ± SD of three independent experiments. Statistical significance was assessed by repeated measurements ANOVA with Bonferroni correction (*p < 0.05, **p < 0.01, ***p < 0.001, ****p < 0.0001).

Since lentivirally transduced HepaRG cells constitutively express Cas9, gene knockout can be easily accomplished by transient transfection of suitable gRNAs. Therefore, we transfected undifferentiated HepaRG^VC^ and HepaRG^-POR^ cells with two sgRNAs targeting *CYB5A* (Fig. 5a), resulting in *CYB5A* single- and *POR/CYB5A* double-knockdown HepaRG cell lines. Characterization following differentiation revealed 50% decrease of CYB5 on mRNA level and ~ 60 to 90% decrease on protein level (Fig. 5b). To analyze the effect of the double-knockdown on CYP-activities we measured these directly in living cells (Fig. 5c). Although all seven CYP-activities appeared to be decreased by 20–40%, only the strongest difference seen for CYP2C8-dependent amodiaquine N-deethylase activity was statistically significant. Most activities were further diminished in the double-knockdown cells, again most profoundly for CYP2C8 activity. Taken together, these and the former NADPH/NADH experiments indicated that several of the human CYP enzyme activities we tested for were markedly influenced by the CYB5 electron donor system and that amodiaquine N-deethylation showed a particularly strong dependence on CYB5 with accordingly less dependence on POR.

### Effects of POR-knockdown on gene expression

**Figure 6:**
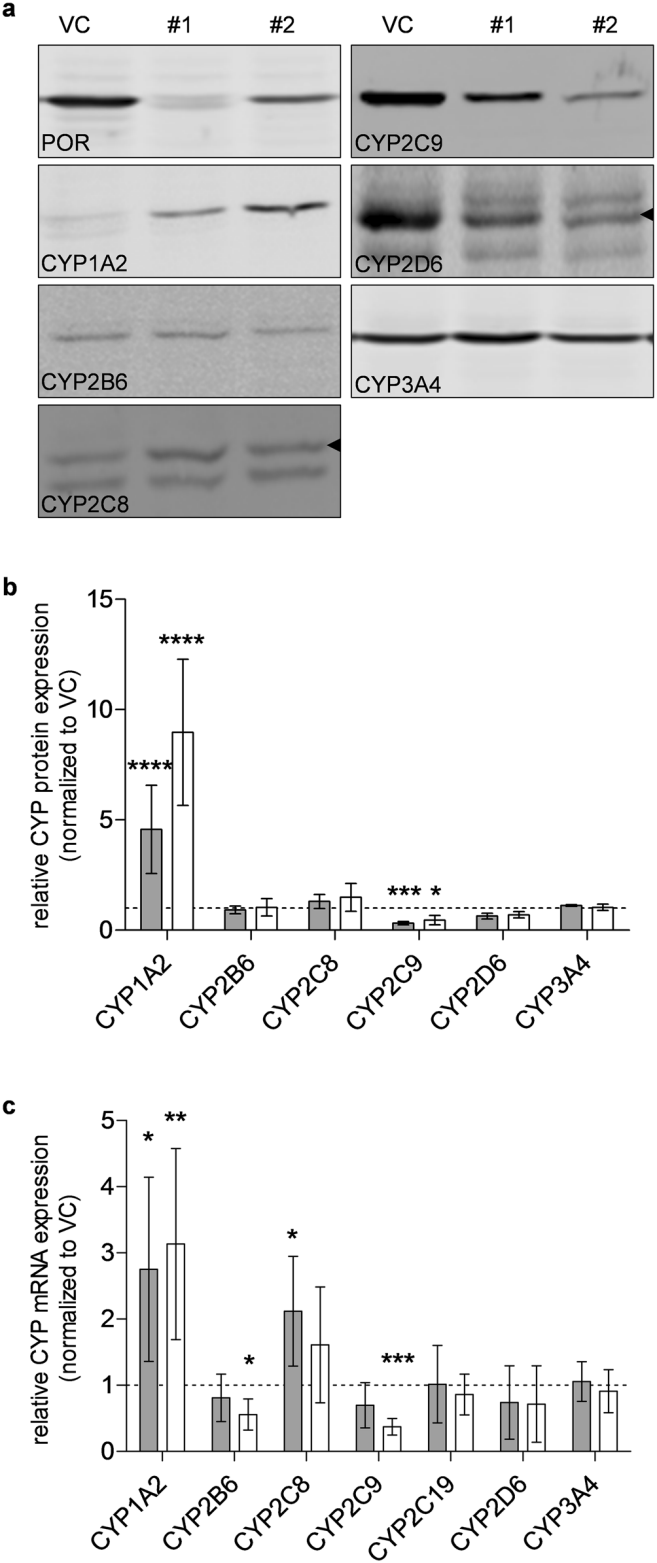
CYP expression analysis in HepaRG^-POR^ microsomes or cell lysates. (**a**) Exemplary Western Blots of microsomal fractions of HepaRG cells transduced with vector control (VC) or sgRNAs POR#1 or POR#2 after differentiation for 3 weeks (see Supplementary Fig. S2 online for full blots). (**b**) Means ± SD of protein expression data of CYP1A2, CYP2B6, CYP2C8, CYP2C9, CYP2D6 and CYP3A4 of 4 independent preparations are shown relative to VC set to 1.0. Statistical significance was assessed by unpaired t-test. (**c**) Gene expression analysis of CYP1A2, CYP2B6, CYP2C8, CYP2C9, CYP2C19, CYP2D6 and CYP3A4 in HepaRG cells transduced with sgRNAs POR#1 and POR#2 and vector control (VC) and differentiated for 2 weeks was performed by qPCR. Data of 6 independent experiments were normalized to the geometric mean of GAPDH, RPLP0 and β-actin. Statistical significance was assessed by paired t-test (*p < 0.05, **p < 0.01, ***p < 0.001, ****p < 0.0001).

The effects of *POR*-knockdown on CYP expression are summarized in Fig. 6. We observed a surprisingly strong increase in CYP1A2 protein level by 4.5- and 9-fold for sgRNAs POR#1 and POR#2, respectively, while CYP2C9 and CYP2D6 were decreased by 50–70% and 30–40%, respectively (Fig. 6a,b). Protein expression of CYPs 2B6, 2C8 and 3A4 was apparently not markedly changed by *POR*-knockdown. Our findings at the protein level were corroborated by measurements of mRNA expression levels, which also showed strongly CYP isoform-dependent effects (Fig. 6c). For CYPs 2B6 and 2C9 mRNA levels were decreased with generally stronger effects seen for sgRNA POR#2, in agreement with the CYP2C9 protein data. The strong induction of CYP1A2 protein was confirmed by an up to 3.13-fold induction of CYP1A2 mRNA. Induced mRNA levels of CYP2C8 as well as unchanged levels of CYP3A4 mRNA were also in good agreement with the protein data.

## Discussion

Here we used CRISPR/Cas9 genome editing in HepaRG cells to study effects of POR and CYB5 on the activity and expression of seven human CYP enzymes. HepaRG cells are usually kept in a proliferative state and then differentiated to hepatocyte-like cells by DMSO^4^. Pilot cell cloning experiments indicated substantial phenotypic heterogeneity among individual cell clones, many of which had lost their differentiation capacity. As we considered it important to maintain cellular characteristics during gene editing, we dismissed the possibility of selecting single clones with heterozygously or homozygously edited target gene as previously described^14, 16^ and instead used lentiviral transduction. Delivery of *Cas9* and a puromycin resistance gene resulted in Cas9-expressing cells (HepaRG^VC^) that closely resemble original HepaRG cells in their phenotypic appearance and the ability to differentiate in 2% DMSO to hepatocyte-like cells with a highly similar CYP expression profile, as shown by close correlation of gene expression and CYP activity profiles (Fig. 1).

Using either one of two different *POR*-specific sgRNAs up to 90% reduction of POR enzyme activity was achieved. The low residual POR presumably derives from cells with heterozygous or no knockout that escaped puromycin selection. Previous *POR*-knockout studies in mammalian cells achieved between ~ 50% up to 100% depending on the technology used^33–40^. The effects of POR reduction on the various CYP-activities differed greatly with CYP2C8-catalyzed amodiaquine N-deethylation being least affected while CYP2C9-catalyzed tolbutamide being the most affected (Fig. 3). Kinetic measurements showed that the reduced CYP activities were generally attributable to reduced V_max_. The comparably low tolbutamide hydroxylase activity may be partially explained by the significantly reduced expression of CYP2C9 in HepaRG^-POR^ microsomes (Fig. 6). Notably, HepaRG cells are homozygous for the decreased function allele CYP2C9*2 that has been described to show lower affinity towards POR^53^.

The small effect of *POR*-knockdown on amodiaquine N-deethylase V_max_ was surprising. Since the activity was strongly inhibited by the specific and potent CYP2C8 inhibitor montelucast^51^, an analytical artefact seemed unlikely. Since commercial bactosomes coexpressing CYP2C8 and different amounts of POR demonstrated a strong influence of POR on the same activity in this different system, a possible explanation was that HepaRG cells support this activity with an alternative redox partner that can compensate for lacking POR. An obvious candidate was CYB5, which is being regenerated by CYB5 reductase and NADH as cofactor. Since POR exhibits only marginal NADH-dependent activity^54^, our observation that all seven CYP-activities could be supported by NADH alone (~15–50% for HepaRG^VC^, Fig. 4) suggests a broad role of CYB5 as electron donor in HepaRG cells. Previous studies in other systems had indicated that CYB5 can act as sole electron donor for several mouse Cyps and at least for human CYP1A1^55^ and that it markedly influences the activity of several drug metabolism activities catalyzed by human CYPs^27, 42, 45, 46^. Our genetic *CYB5A* single- and *POR/CYB5A*-double knockout experiments directly confirmed the effect of CYB5 on some CYP-activities and on amodiaquine N-deethylation in particular (Fig. 5). With the possible exception of CYPs 1A2 and 2D6, the effect of *CYB5A*-knockdown was even greater in the *POR*-knockdown cells, suggesting that the CYB5/CYB5 reductase system compensates in part for lacking POR, probably due to unique but overlapping interaction sites for CYB5 and POR^43, 56^. The strongest impact of *CYB5A*-knockdown on amodiaquine N-deethylation, especially in the double-knockdown cells, further demonstrated a particularly strong role of CYB5 for CYP2C8. Although there is evidence for CYB5 showing preferences for certain cytochrome P450 isozymes as well as particular reactions, we are not aware of any studies that included CYP2C8^57^. Interestingly, we found that HepaRG cells are homozygous for CYP2C8*3, a relatively common variant reported to show altered interaction properties with POR and CYB5 in vitro^58, 59^.

One surprising result of the liver-specific deletion of *Por* in mice were the generally increased Cyp expression profiles, which suggested some compensatory mechanisms that have not yet been clarified nor have they been confirmed in a human cell culture system to our knowledge^24, 25^. In HepaRG^-POR^ cells we observed differential effects with CYP1A2 being severalfold induced while CYP2C9 was severalfold decreased, with data on mRNA and protein level being well in concordance (Fig. 6). The reasons for these profound differential effects on CYP expression as well as for the apparent species differences remain speculative. Due to the wide differences in CYP genes between mice and humans, which concern number, sequence and structure, it is difficult to directly compare gene regulatory effects. As a cautionary note it should also be kept in mind that HepaRG is an immortalized cell line that appears to reflect many aspects of human drug metabolism correctly but may also differ in certain aspects of dynamic gene regulation in comparison to other cell models or the liver as an organ.

In summary, using lentiviral transduction we have created a HepaRG cell line that constitutively expresses Cas9 and that retains the ability to differentiate into hepatocyte-like cells with cytochrome P450 expression and activity profiles highly similar to those of the parent cell line. Genetic knockout of *POR* and *CYB5A* resulted in differential, CYP isoform-dependent effects on CYP expression and activities. Our data support a general role of CYB5 for drug metabolism in HepaRG cells as previously observed in other human drug metabolism systems. The Cas9-expressing HepaRG^VC^ cells should be a versatile tool to study the influence of diverse genes on drug metabolism and other hepatic functions in a metabolically competent human hepatic cell line.

## Methods

### HepaRG cell culture and DNA sequencing

HepaRG cells (batch HPR101007, passage no. 12)^4^ were obtained from Biopredic International (Rennes, FR) and cultured as described^9^. For sequencing of *POR*, *CYP1A2*, *CYP2D6* and *CYP2C8*, genomic DNA isolated using QIAamp DNA mini Kit (Qiagen, Hilden, USA) of HepaRG cells was amplified by PCR^31, 60–62^. Sanger sequencing was performed by Microsynth Seqlab (Göttingen, DE). *POR* and *CYP* allelic annotation was done according to Pharmacogene Variation Consortium (PharmVar) at www.PharmVar.org^47, 48^. OpenArray plates with a QuantStudio 12 k flex system (ThermoFisher, Waltham, USA) equipped with pre-desigend TaqMan assays (ThermoFisher) (Table 1) were used to genotype the most important variants of *CYP2B6*, *2C9*, *2C19* and *3A4/5*. In brief, 3 µL DNA (50 ng/µL), mixed with OpenArray Genotyping MasterMix was applied on the OpenArray plate using the AccuFill Loader (ThermoFisher). The plate was sealed within 90 s and placed into the real-time PCR machine. The PCR was performed using the genotyping mode. Genotypes were called using the Genotyper Software (ThermoFisher).

### CRISPR/Cas9 genome editing

Single guide RNAs (sgRNA) targeting two distinct regions of the human *POR* as well as the human *CYB5* gene were designed using the online tool CHOPCHOP^63^. The sgRNA sequences were: 5′-TCGTGGGTCTCCTAACCTACtgg-3′ (sgRNA POR#1), 5′-GTGTTCTACGGCTCCCAGAcgg-3′ (sgRNA POR#2)^39^ 5′-AATCGTACACCTTGTGGTGCagg-3′ (sgRNA CYB5#1) and 5′-TCGGGCACTCTACAGATGCCagg-3′ (sgRNA CYB5#2) (PAM sequences shown in lower case letters). sgRNAs for *POR* were cloned into the *BsmB*I site of the lentiCRISPRv2 lentiviral vector (a gift from Feng Zhang; Addgene #52961) according to Shalem et al.^64^, the empty lentiCRISPRv2 vector was used as vector control (VC). Correct insertion was verified by Sanger sequencing. Lentiviruses carrying the plasmid coding for Cas9 and the sgRNA were generated in HEK293T cells by co-transfection of the packaging plasmids psPAX.2 (a gift from Didier Trono; Addgene #12260) and pCMV-VSV-G (a gift from Bob Weinberg; Addgene # 8454). Supernatants containing lentiviral particles were harvested 48 h post-transfection. HepaRG cells were transduced in OptiMEM (Gibco, Carslbad, USA) containing 10 μg/mL polybrene. The resulting cell lines express Cas9 (HepaRG^VC^) as well as a sgRNA (HepaRG^-POR#1^, HepaRG^-POR#2^). Further selection was done with puromycin (5 μg/mL). sgRNAs for CYB5 were delivered into undifferentiated Cas9 expressing HepaRG^-POR#1^, HepaRG^-POR#2^ and HepaRG^VC^ by reverse transfection of 30 nM sgRNAs with Lipofectamine RNAiMax (ThermoFisher). T7 endonuclease 1 (T7E1) assay was used to confirm CRISPR/Cas9 induced gene editing by PCR amplification using specific primers of sgRNA target regions (Supplementary Table S1 online) with following denaturation and reannealing (cycle conditions: 95 °C for 10 min, ramp down to 85 °C at 2 °C/s, ramp down to 25 °C at 0.3 °C/s). T7E1 digestion (NEB, Ipswich, USA) was performed at 37 °C for 60 min. The digested fragments were analyzed on a 1% agarose gel.

### Cytochrome C reduction assay

Cytochrome P450 reductase activity was determined in 15–30 µg of microsomal protein using the cytochrome C reduction assay as described elsewhere^31, 65^.

### Microsomes and cytochrome P450 activity measurements

For microsome preparation HepaRG cells were cultivated and differentiated in T175 flasks, harvested and mechanically disrupted. Bactosomes containing human CYP2C8 (CYP/EZ017, CYP/EZ047), CYP2C9 (CYP/EZ019, CYP/EZ006) and CYP3A4 (CYP/EZ002, CYP/EZ010) and human POR in high and low concentrations coexpressed in *Escherichia coli* were purchased from Cypex Ltd. (Dundee, UK). For determination of CYP enzyme activities in microsomal preparations a liquid chromatography with tandem mass spectrometry (LC–MS/MS) substrate cocktail assay was used essentially as described elsewhere^12, 66^. For determination of CYP enzyme activities in cell culture supernatant, the cells were incubated with the substrate cocktail for 3 h at 37 °C in the incubator. Metabolites were quantified in supernatants as described^66^. For microsomal kinetic experiments, 2.5—20 µg of microsomes in 0.1 N potassium phosphate buffer (pH 7.4) were incubated with substrates and/or inhibitors at the indicated concentrations, and NADPH-regenerating system for 20–30 min depending on the substrate. For direct comparison of NADPH- and NADH-driven metabolism, the cofactors were used as pure substances at 1 mM concentration with substrate cocktail as described above.

### Flow cytometric and immunoblot analysis

For flow cytometric analysis of Cas9 in HepaRG cells as primary antibody mouse monoclonal anti-CRISPR-Cas9 antibody (ab191468, abcam, Cambridge, UK) and as secondary antibody Alexa Fluor 488 polyclonal goat anti-mouse IgG H&L antibody (ab150113, abcam) was used. Western blot (WB) analysis in 20 µg of microsomal protein was performed as described^33^. For the analysis of Cas9, POR, CYB5 and CYPs 1A2, 2B6, 2C8, 2C9, 2D6 and 3A4 the following antibodies were used: mouse monoclonal anti-CRISPR-Cas9 antibody; rabbit anti-human POR polyclonal antibody raised against affinity-purified rat POR (U. M. Zanger and U. A. Meyer, University of Basel, CH, unpublished data); mouse anti-human CYB5 monoclonal antibody (sc-130311, Santa Cruz Biotechnology, Dallas, USA); mouse monoclonal anti-human CYP1A2 antibody (clone 26-7-5, a kind gift of Frank Gonzalez, Bethesda, USA); mouse monoclonal anti-human CYP2B6 (#458326 Gentest Corp., Woburn, USA); polyclonal rabbit anti-human CYP2C8 (#Hu-A004 Puracyp Inc., Carlsbad, USA); polyclonal rabbit anti-human CYP2C9 (RDI-Cyp2C9abr, Research diagnosics inc., Flanders, USA); monoclonal mouse anti-human CYP2D6 (Mab 114^67^); and a rabbit polyclonal anti-human CYP3A4 antibody (#458234 Gentest Corp.). Secondary antibodies were fluorescence labeled goat anti-mouse IRDye 680 and 800 and goat anti-rabbit IRDye 680 and 800 (LI-COR Biosciences GmbH, Bad Homburg, DE), and visualized using the IR imaging system Odyssey (LI-COR Biosciences GmbH).

### Quantitative real-time PCR for gene expression analysis

100–200 ng of total RNA (isolated using RNeasy Plus Kit (Qiagen)) was reverse transcribed with TaqMan Reverse Transcription Reagents (Applied Biosystems, Foster City, USA). cDNA was preamplified using TaqMan PreAmp Mastermix (Applied Biosystems) and expression of 82 genes was quantified using the Biomark HD system (Fluidigm, San Francisco, USA) with a 48:48 Dynamic Array Chip (Fluidigm). Expression levels were determined in triplicates and normalized to the geometric mean of glyceraldehyde-3-phosphate dehydrogenase (GAPDH), RPLP0 and β-actin expression^68^ and relatively quantified using the ΔΔct method.

### Statistical analyses

Statistical analyses were performed using Graphpad Prism V5 software (GraphPad, San Diego, USA). Spearman’s correlation coefficients and corresponding tests were used to assess the associations between wildtype HepaRG and HepaRG^VC^ on CYP activity and gene expression level. Repeated measurements ANOVA with Bonferroni correction or appropriate paired/unpaired t-test statistics were applied on log-transformed data, significance level was set to *p* < 0.05. Results are shown as means ± standard deviation (SD) or with 95% confidence intervals (95% CI), as appropriate. Kinetic parameters K_M_ and V_max_ were determined by Michaelis–Menten model or by substrate inhibition model, inhibition parameters IC_50_ and K_i_ were determined by one site competition model using Graphpad Prism V5 software (GraphPad). Internal clearance (Cl_int_) was calculated using the following equation: ${\mathrm{Cl}}_{\mathrm{int}}=\frac{{\mathrm{V}}_{\mathrm{max}}}{{\mathrm{K}}_{\mathrm{M}}}$

## Supplementary Information

Supplementary Information.

## References

[1] Karlgren M, Simoff I, Keiser M, Oswald S, Artursson P (2018). CRISPR-Cas9: a new addition to the drug metabolism and disposition tool box. Drug Metab. Dispos..

[2] Pankowicz FP, Jarrett KE, Lagor WR, Bissig K-D (2017). CRISPR/Cas9: at the cutting edge of hepatology. Gut.

[3] Godoy P, Hewitt NJ, Albrecht U, Andersen ME, Ansari N, Bhattacharya S (2013). Recent advances in 2D and 3D in vitro systems using primary hepatocytes, alternative hepatocyte sources and non-parenchymal liver cells and their use in investigating mechanisms of hepatotoxicity, cell signaling and ADME. Arch. Toxicol..

[4] Gripon P, Rumin S, Urban S, Le Seyec J, Glaise D, Cannie I (2002). Infection of a human hepatoma cell line by hepatitis B virus. Proc. Natl. Acad. Sci. USA.

[5] Rogue A, Lambert C, Spire C, Claude N, Guillouzo A (2012). Interindividual variability in gene expression profiles in human hepatocytes and comparison with HepaRG cells. Drug Metab. Dispos..

[6] Kanebratt KP, Andersson TB (2008). Evaluation of HepaRG cells as an in vitro model for human drug metabolism studies. Drug Metab. Dispos..

[7] Andersson TB, Kanebratt KP, Kenna JG (2012). The HepaRG cell line: a unique in vitro tool for understanding drug metabolism and toxicology in human. Expert Opin. Drug Metab. Toxicol..

[8] Rubin K, Janefeldt A, Andersson L, Berke Z, Grime K, Andersson TB (2015). HepaRG cells as human-relevant in vitro model to study the effects of inflammatory stimuli on cytochrome P450 isoenzymes. Drug Metab. Dispos..

[9] Klein M, Thomas M, Hofmann U, Seehofer D, Damm G, Zanger UM (2015). A systematic comparison of the impact of inflammatory signaling on absorption, distribution, metabolism, and excretion gene expression and activity in primary human hepatocytes and HepaRG cells. Drug Metab. Dispos..

[10] Tolosa L, Gómez-Lechón MJ, Jiménez N, Hervás D, Jover R, Donato MT (2016). Advantageous use of HepaRG cells for the screening and mechanistic study of drug-induced steatosis. Toxicol. Appl. Pharmacol..

[11] Tanner N, Kubik L, Luckert C, Thomas M, Hofmann U, Zanger UM (2018). Regulation of drug metabolism by the interplay of inflammatory signaling, steatosis, and xeno-sensing receptors in HepaRG cells. Drug Metab. Dispos..

[12] Kugler N, Klein K, Zanger UM (2020). MiR-155 and other microRNAs downregulate drug metabolizing cytochromes P450 in inflammation. Biochem. Pharmacol..

[13] Kennedy EM, Bassit LC, Mueller H, Kornepati AVR, Bogerd HP, Nie T (2015). Suppression of hepatitis B virus DNA accumulation in chronically infected cells using a bacterial CRISPR/Cas RNA-guided DNA endonuclease. Virology.

[14] Bucher S, Le Guillou D, Allard J, Pinon G, Begriche K, Tête A (2018). Possible involvement of mitochondrial dysfunction and oxidative stress in a cellular model of NAFLD progression induced by benzoapyrene/ethanol CoExposure. Oxid. Med. Cell Longev..

[15] Namineni S, O'Connor T, Faure-Dupuy S, Johansen P, Riedl T, Liu K (2020). A dual role for hepatocyte-intrinsic canonical NF-κB signaling in virus control. J. Hepatol..

[16] Wei Y, Huai C, Zhou C, Gao Y, Chen L, Zhou W (2020). A methylation functional detection hepatic cell system validates correlation between DNA methylation and drug-induced liver injury. Pharmacogenom. J..

[17] Pandey AV, Flück CE (2013). NADPH P450 oxidoreductase: structure, function, and pathology of diseases. Pharmacol. Ther..

[18] Porter TD (2012). New insights into the role of cytochrome P450 reductase (POR) in microsomal redox biology. Acta Pharm. Sin. B..

[19] Huang R, Zhang M, Rwere F, Waskell L, Ramamoorthy A (2015). Kinetic and structural characterization of the interaction between the FMN binding domain of cytochrome P450 reductase and cytochrome c. J. Biol. Chem..

[20] Barnaba C, Ramamoorthy A (2018). Picturing the membrane-assisted choreography of cytochrome P450 with lipid nanodiscs. ChemPhysChem.

[21] Gentry KA, Anantharamaiah GM, Ramamoorthy A (2019). Probing protein–protein and protein–substrate interactions in the dynamic membrane-associated ternary complex of cytochromes P450, b(5), and reductase. Chem. Commun. (Camb).

[22] Shen AL, O'Leary KA, Kasper CB (2002). Association of multiple developmental defects and embryonic lethality with loss of microsomal NADPH-cytochrome P450 oxidoreductase. J. Biol. Chem..

[23] Otto DME, Henderson CJ, Carrie D, Davey M, Gundersen TE, Blomhoff R (2003). Identification of novel roles of the cytochrome P450 system in early embryogenesis: effects on vasculogenesis and retinoic acid homeostasis. Mol. Cell Biol..

[24] Gu J, Weng Y, Zhang Q-Y, Cui H, Behr M, Wu L (2003). Liver-specific deletion of the NADPH-cytochrome P450 reductase gene: impact on plasma cholesterol homeostasis and the function and regulation of microsomal cytochrome P450 and heme oxygenase. J. Biol. Chem..

[25] Henderson CJ, Otto DME, Carrie D, Magnuson MA, McLaren AW, Rosewell I, Wolf CR (2003). Inactivation of the hepatic cytochrome P450 system by conditional deletion of hepatic cytochrome P450 reductase. J. Biol. Chem..

[26] Finn RD, McLaren AW, Carrie D, Henderson CJ, Wolf CR (2007). Conditional deletion of cytochrome P450 oxidoreductase in the liver and gastrointestinal tract: a new model for studying the functions of the P450 system. J. Pharmacol. Exp. Ther..

[27] Henderson CJ, McLaughlin LA, Wolf CR (2013). Evidence that cytochrome b5 and cytochrome b5 reductase can act as sole electron donors to the hepatic cytochrome P450 system. Mol. Pharmacol..

[28] Yamazaki H, Nakano M, Gillam EM, Bell LC, Guengerich FP, Shimada T (1996). Requirements for cytochrome b5 in the oxidation of 7-ethoxycoumarin, chlorzoxazone, aniline, and N-nitrosodimethylamine by recombinant cytochrome P450 2E1 and by human liver microsomes. Biochem. Pharmacol..

[29] Guengerich FP (2001). Common and uncommon cytochrome P450 reactions related to metabolism and chemical toxicity. Chem. Res. Toxicol..

[30] Hart SN, Wang S, Nakamoto K, Wesselman C, Li Y, Zhong X-b (2008). Genetic polymorphisms in cytochrome P450 oxidoreductase influence microsomal P450-catalyzed drug metabolism. Pharmacogenet. Genom..

[31] Gomes AM, Winter S, Klein K, Turpeinen M, Schaeffeler E, Schwab M, Zanger UM (2009). Pharmacogenomics of human liver cytochrome P450 oxidoreductase: multifactorial analysis and impact on microsomal drug oxidation. Pharmacogenomics.

[32] Connick JP, Reed JR, Backes WL (2018). Characterization of interactions among CYP1A2, CYP2B4, and NADPH-cytochrome P450 reductase: identification of specific protein complexes. Drug Metab. Dispos..

[33] Feidt DM, Klein K, Nüssler A, Zanger UM (2009). RNA-interference approach to study functions of NADPH: cytochrome P450 oxidoreductase in human hepatocytes. Chem. Biodivers..

[34] Porter TD, Banerjee S, Stolarczyk EI, Zou L (2011). Suppression of cytochrome P450 reductase (POR) expression in hepatoma cells replicates the hepatic lipidosis observed in hepatic POR-null mice. Drug Metab. Dispos..

[35] Sundberg CD, Hankinson O (2019). A CRISPR/Cas9 whole-genome screen identifies genes required for aryl hydrocarbon receptor-dependent induction of functional CYP1A1. Toxicol. Sci..

[36] Reed L, Jarvis IWH, Phillips DH, Arlt VM (2019). Deletion of cytochrome P450 oxidoreductase enhances metabolism and DNA adduct formation of benzoapyrene in Hepa1c1c7 cells. Mutagenesis.

[37] Hunter FW, Devaux JBL, Meng F, Hong CR, Khan A, Tsai P (2019). Functional CRISPR and shRNA screens identify involvement of mitochondrial electron transport in the activation of evofosfamide. Mol. Pharmacol..

[38] Hunter FW, Young RJ, Shalev Z, Vellanki RN, Wang J, Gu Y (2015). Identification of P450 oxidoreductase as a major determinant of sensitivity to hypoxia-activated prodrugs. Cancer Res..

[39] Rezende F, Prior K-K, Löwe O, Wittig I, Strecker V, Moll F (2017). Cytochrome P450 enzymes but not NADPH oxidases are the source of the NADPH-dependent lucigenin chemiluminescence in membrane assays. Free Radic. Biol. Med..

[40] Su J, Gu Y, Pruijn FB, Smaill JB, Patterson AV, Guise CP, Wilson WR (2013). Zinc finger nuclease knock-out of NADPH: cytochrome P450 oxidoreductase (POR) in human tumor cell lines demonstrates that hypoxia-activated prodrugs differ in POR dependence. J. Biol. Chem..

[41] Yoo S-E, Yi M, Kim W-Y, Cho S-A, Lee SS, Lee S-J, Shin J-G (2019). Influences of cytochrome b5 expression and its genetic variant on the activity of CYP2C9, CYP2C19 and CYP3A4. Drug Metab. Pharmacokinet..

[42] Yamazaki H, Nakamura M, Komatsu T, Ohyama K, Hatanaka N, Asahi S (2002). Roles of NADPH-P450 reductase and apo- and holo-cytochrome b5 on xenobiotic oxidations catalyzed by 12 recombinant human cytochrome P450s expressed in membranes of Escherichia coli. Protein Express. Purif..

[43] Zhang H, Im S-C, Waskell L (2007). Cytochrome b5 increases the rate of product formation by cytochrome P450 2B4 and competes with cytochrome P450 reductase for a binding site on cytochrome P450 2B4. J. Biol. Chem..

[44] Ahuja S, Jahr N, Im S-C, Vivekanandan S, Popovych N, Le Clair SV (2013). A model of the membrane-bound cytochrome b5-cytochrome P450 complex from NMR and mutagenesis data. J. Biol. Chem..

[45] Henderson CJ, McLaughlin LA, Finn RD, Ronseaux S, Kapelyukh Y, Wolf CR (2014). A role for cytochrome b5 in the In vivo disposition of anticancer and cytochrome P450 probe drugs in mice. Drug Metab. Dispos..

[46] Henderson CJ, McLaughlin LA, Scheer N, Stanley LA, Wolf CR (2015). Cytochrome b5 is a major determinant of human cytochrome P450 CYP2D6 and CYP3A4 activity in vivo. Mol. Pharmacol..

[47] Gaedigk A, Ingelman-Sundberg M, Miller NA, Leeder JS, Whirl-Carrillo M, Klein TE (2018). The pharmacogene variation (PharmVar) consortium: incorporation of the human cytochrome P450 (CYP) allele nomenclature database. Clin. Pharmacol. Ther..

[48] Gaedigk A, Sangkuhl K, Whirl-Carrillo M, Twist GP, Klein TE, Miller NA (2019). The evolution of PharmVar. Clin. Pharmacol. Ther..

[49] Jackson JP, Li L, Chamberlain ED, Wang H, Ferguson SS (2016). Contextualizing hepatocyte functionality of cryopreserved HepaRG cell cultures. Drug Metab. Dispos..

[50] Laurent V, Fraix A, Montier T, Cammas-Marion S, Ribault C, Benvegnu T (2010). Highly efficient gene transfer into hepatocyte-like HepaRG cells: new means for drug metabolism and toxicity studies. Biotechnol. J..

[51] Walsky RL, Obach RS, Gaman EA, Gleeson J-PR, Proctor WR (2005). Selective inhibition of human cytochrome P4502C8 by montelukast. Drug Metab. Dispos..

[52] Li D, Mackowiak B, Brayman TG, Mitchell M, Zhang L, Huang S-M, Wang H (2015). Genome-wide analysis of human constitutive androstane receptor (CAR) transcriptome in wild-type and CAR-knockout HepaRG cells. Biochem. Pharmacol..

[53] Crespi CL, Miller VP (1997). The R144C change in the CYP2C9*2 allele alters interaction of the cytochrome P450 with NADPH:cytochrome P450 oxidoreductase. Pharmacogenetics.

[54] Döhr O, Paine MJ, Friedberg T, Roberts GC, Wolf CR (2001). Engineering of a functional human NADH-dependent cytochrome P450 system. Proc. Natl. Acad. Sci. USA.

[55] Stiborová M, Indra R, Moserová M, Šulc M, Hodek P, Frei E (2016). NADPH- and NADH-dependent metabolism of and DNA adduct formation by benzoapyrene catalyzed with rat hepatic microsomes and cytochrome P450 1A1. Monatsh. Chem..

[56] Waskell, L., Kim, J.-J. P. Electron transfer partners of cytochrome P450. In *Cytochrome P450*. 4th ed, 33–68 (ed. Ortiz de Montellano PR) Structure, mechanism, and biochemistry.

[57] Gentry KA, Zhang M, Im S-C, Waskell L, Ramamoorthy A (2018). Substrate mediated redox partner selectivity of cytochrome P450. Chem. Commun. (Camb).

[58] Arnold WR, Zelasko S, Meling DD, Sam K, Das A (2019). Polymorphisms of CYP2C8 alter first-electron transfer kinetics and increase catalytic uncoupling. Int. J. Mol. Sci..

[59] Kaspera R, Naraharisetti SB, Evangelista EA, Marciante KD, Psaty BM, Totah RA (2011). Drug metabolism by CYP2C8.3 is determined by substrate dependent interactions with cytochrome P450 reductase and cytochrome b5. Biochem. Pharmacol..

[60] Yeo C-W, Lee S-J, Lee SS, Bae SK, Kim E-Y, Shon J-H (2011). Discovery of a novel allelic variant of CYP2C8, CYP2C8*11, in Asian populations and its clinical effect on the rosiglitazone disposition in vivo. Drug Metab. Dispos..

[61] Geng T, Zhang XY, Wang L, Wang H, Shi X, Kang L (2016). Genetic polymorphism analysis of the drug-metabolizing enzyme CYP1A2 in a Uyghur Chinese population: a pilot study. Xenobiotica.

[62] Raimundo S, Toscano C, Klein K, Fischer J, Griese E-U, Eichelbaum M (2004). A novel intronic mutation, 2988GA, with high predictivity for impaired function of cytochrome P450 2D6 in white subjects. Clin. Pharmacol. Ther..

[63] Labun K, Montague TG, Krause M, Torres Cleuren YN, Tjeldnes H, Valen E (2019). CHOPCHOP v3: expanding the CRISPR web toolbox beyond genome editing. Nucleic Acids Res..

[64] Shalem O, Sanjana NE, Hartenian E, Shi X, Scott DA, Mikkelson T (2014). Genome-scale CRISPR-Cas9 knockout screening in human cells. Science.

[65] Guengerich FP, Martin MV, Sohl CD, Cheng Q (2009). Measurement of cytochrome P450 and NADPH-cytochrome P450 reductase. Nat. Protoc..

[66] Feidt DM, Klein K, Hofmann U, Riedmaier S, Knobeloch D, Thasler WE (2010). Profiling induction of cytochrome p450 enzyme activity by statins using a new liquid chromatography-tandem mass spectrometry cocktail assay in human hepatocytes. Drug Metab. Dispos..

[67] Zanger UM, Fischer J, Raimundo S, Stüven T, Evert BO, Schwab M, Eichelbaum M (2001). Comprehensive analysis of the genetic factors determining expression and function of hepatic CYP2D6. Pharmacogenetics..

[68] Vandesompele J, de Preter K, Pattyn F, Poppe B, van Roy N, de Paepe A, Speleman F (2002). Accurate normalization of real-time quantitative RT-PCR data by geometric averaging of multiple internal control genes. Genome Biol..

