# Supplementary material for: Differential effects on human cytochromes P450 by CRISPR/Cas9-induced genetic knockout of cytochrome P450 reductase and cytochrome b5 in HepaRG cells
Source: Sci Rep. 2021 Jan 13;11:1000. doi: 10.1038/s41598-020-79952-1 (PMC7806635; doi:10.1038/s41598-020-79952-1)
Supplement: Supplementary file 1 — Supplementary Information. [file 41598_2020_79952_MOESM1_ESM.pdf]

Differential effects on human cytochromes P450 by CRISPR/Cas9-induced genetic knockout of cytochrome P450 reductase and cytochrome b5 in HepaRG cells

Tamara Heintze[1,2], Kathrin Klein[1,2], Ute Hofmann[1,2], Ulrich M. Zanger[1,2*]

***Correspondence:**

uli.zanger@ikp-stuttgart.de

Supplementary Information:

Supplementary Figure S1: Full length western blot of Fig. 1b

Supplementary Figure S2: Full length western blots of Fig. 6a

Supplementary Table S1: Sequences of primers used for PCR amplification of sgRNA target sites for T7E1 digest

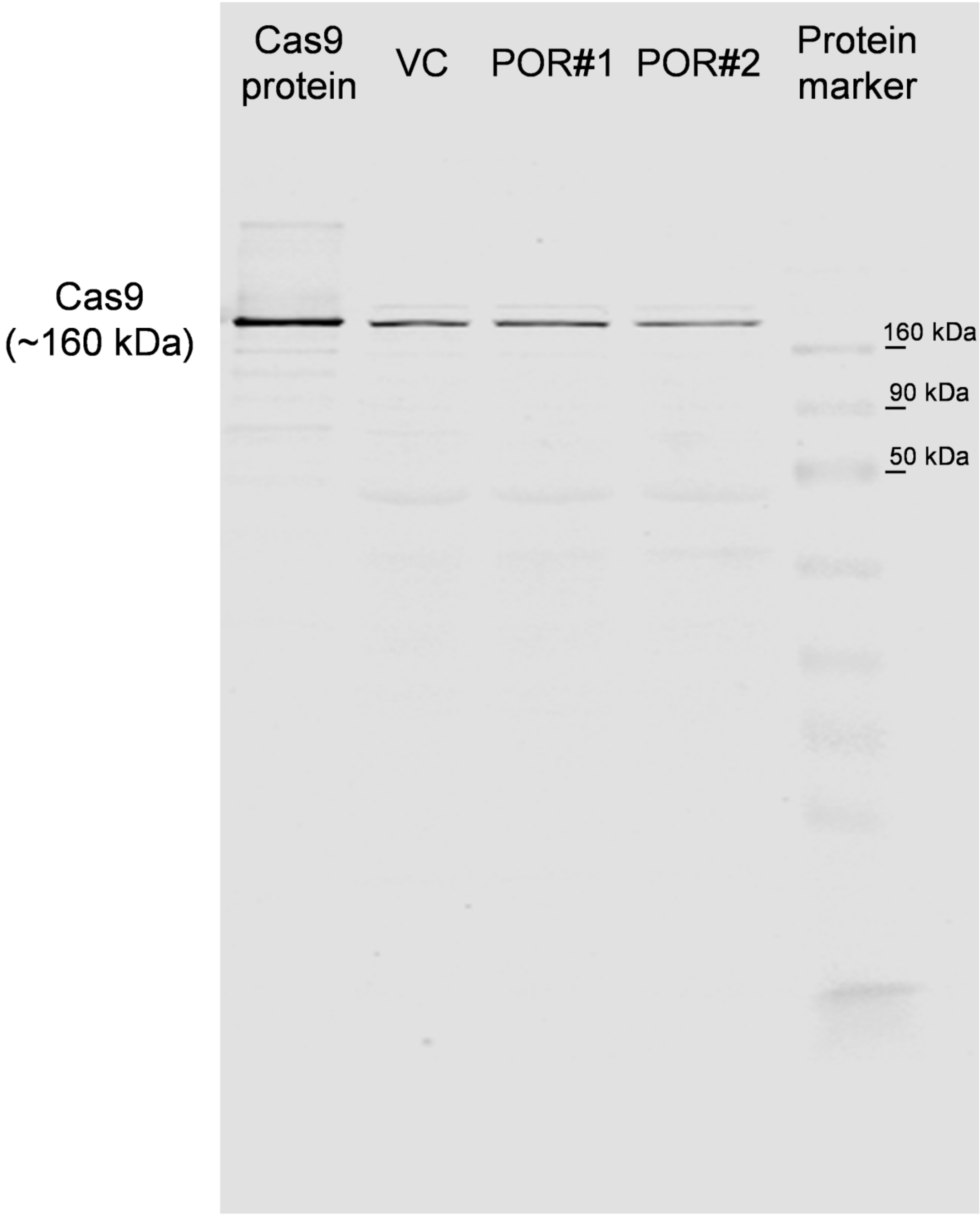

Supplementary Figure S1: Full length western blot of Fig. 1b. Analysis of Cas9 expression in lysates of undifferentiated HepaRG^{VC}, HepaRG $^{–POR\#1}$ and HepaRG$^{-POR\#2}$, Cas9 protein as positive control. Protein Marker: Chameleon^{TM} Duo LI-COR.

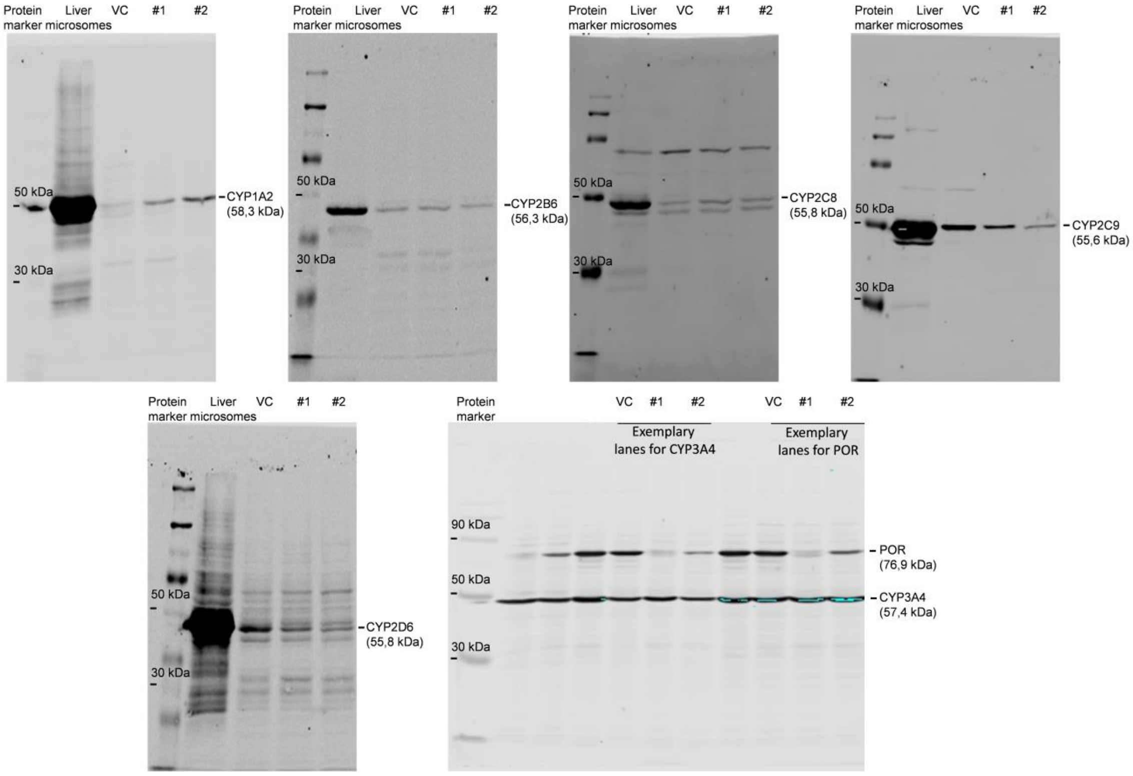

Supplementary Figure S2: Full length western blots of Fig. 6a: Exemplary Western Blots of microsomal fractions of HepaRG cells transduced with vector control (VC) or sgRNAs POR#1 or POR#2 after differentiation for three weeks. CYP expression in liver microsomes was analysed as positive control. Protein Marker: Chameleon^{TM} Duo LI-COR.

Supplementary Table S1: Sequences of primer used for PCR amplification of sgRNA target sites for T7E1 digest.

Primer name	Sequence	Function
**POR#1 fwd**	5′-GGATGTTCCAGCACACTGAGA-3′	Primer for T7E1 digest of sgRNA POR#1 target site
**POR#1 rev**	5′-CCAAGAGTCACCCCAAAATGC-3′	
**POR#2 fwd**	5′-TGTGTGAGATTGCCTTGGTGA-3′	Primer for T7E1 digest of sgRNA POR#2 target site
**POR#2 rev**	5′-ACGGGAAGGCAACTTCCGA-3′	
**POR off-target#1 fwd**	5′-GACTGTCAGCGGGTGGAAAA-3′	Primer for T7E1 digest of predicted off-target site #1 of sgRNA POR#2
**POR off-target#1 rev**	5′-TGAGGAGGTTTCCTGGTGGA-3′	
**POR off-target#2 fwd**	5′-TCCTACGGGTTGTGTTGAGC-3′	Primer for T7E1 digest of predicted off-target site #2 of sgRNA POR#2
**POR off-target#2 rev**	5′-TGGCTGATTAACCAGGTGCC-3′	
**POR off-target#3 fwd**	5′-TGGCATAGTCCATAAGCTTGC-3′	Primer for T7E1 digest of predicted off-target site #3 of sgRNA POR#2
**POR off-target#3 rev**	5′-ACAACAGCTGCTAAGGGTCC-3′	
